# Golden Rules of Dietetics

**Published:** 1909-05

**Authors:** 


**Golden Rules of Dietetics.** By **A. L. Benedict,** A.M., M.D., Author of Practical Dietetics. 12 mo, pp. 407. St. Louis: C. V. Mosby Co. (Cloth, $3.00.)

This is an excellent presentation of the general principles of the science of dietetics in the form of terse rules for the guidance of the general practitioner. The author has not bound himself by any hard and fast rules, but has given the subject the broadest consideration in all its phases and has done into a book the best there is in present day dietetics. He has avoided that purely scholastic research work which clutters so many of the smaller scientific works, and while he has drawn from the experience and work of others the greater part of the book is the result of his own large experience. He gives his reader a clear insight into the true significance of dietetics.

Beginning with the composition of the body the author takes up the daily and the standard requirements of the human body and the standard diets in life. Waste and predigestion of foods are given careful consideration; much more so than is usual even in larger works on the same subject. An entire chapter is devoted to deleterious food stuffs and another to the general hygiene of eating. The diet lists are extremely valuable and will be found of assistance, as will the diets in various diseases, all of which are very complete, even to what a casual observer might consider inconsequential details. The feeding in surgical operations and emergencies is a chapter which is full to overflowing of useful directions, and the recipes for the preparation of food for the invalid will be found of practical value. The book is not pretentious; it is rather more; it is absolutely useful.

